# Internet of Things: Evolution, Concerns and Security Challenges

**DOI:** 10.3390/s21051809

**Published:** 2021-03-05

**Authors:** Parushi Malhotra, Yashwant Singh, Pooja Anand, Deep Kumar Bangotra, Pradeep Kumar Singh, Wei-Chiang Hong

**Affiliations:** 1 Department of Computer Science and Information Technology, Central University of Jammu, Bagla J&K 181143, India; malhotra.parushi@gmail.com (P.M.); yashwant.csit@cujammu.ac.in (Y.S.); poojaanand892@gmail.com (P.A.); 2 Department of Higher Education, J&K Govt., Jammu 180001, India; deepbangotra.ap@gmail.com; 3 ABES Engineering College, Ghaziabad 201309, India; 4 Department of Information Management, Oriental Institute of Technology, New Taipei City 22064, Taiwan

**Keywords:** Internet of Things (IoT), machine learning, deep learning, intrusion detection system, wireless sensor network, testbed

## Abstract

The escalated growth of the Internet of Things (IoT) has started to reform and reshape our lives. The deployment of a large number of objects adhered to the internet has unlocked the vision of the smart world around us, thereby paving a road towards automation and humongous data generation and collection. This automation and continuous explosion of personal and professional information to the digital world provides a potent ground to the adversaries to perform numerous cyber-attacks, thus making security in IoT a sizeable concern. Hence, timely detection and prevention of such threats are pre-requisites to prevent serious consequences. The survey conducted provides a brief insight into the technology with prime attention towards the various attacks and anomalies and their detection based on the intelligent intrusion detection system (IDS). The comprehensive look-over presented in this paper provides an in-depth analysis and assessment of diverse machine learning and deep learning-based network intrusion detection system (NIDS). Additionally, a case study of healthcare in IoT is presented. The study depicts the architecture, security, and privacy issues and application of learning paradigms in this sector. The research assessment is finally concluded by listing the results derived from the literature. Additionally, the paper discusses numerous research challenges to allow further rectifications in the approaches to deal with unusual complications.

## 1. Introduction

**Figure 1:**
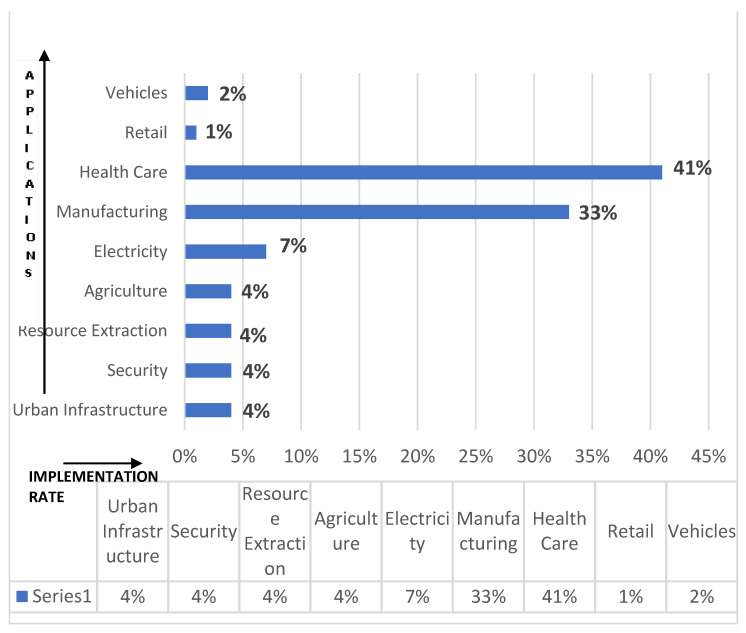
Applications of Internet of Things (IoT) with practical Implementations [1].

The rapid escalation in numerous technological aspects of wireless sensor networks (WSN), mobile communication, radio-frequency identification (RFID), and various lightweight protocols have endorsed the concept of the Internet of Things. The core conviction of IoT revolves around the dynamic interconnection of billions of different units or entities in an ecosystem driving either in a wired or a wireless fashion via the assistance of intelligent sensors, actuators, and other components. These components mesh with each other to yield the state of things and thus, providing extensive benefits and comforts to humans. Numbers stipulate that the IoT market has reached a mark of approximately 200 billion in 2020, starting with just 2 billion in 2006 [1]. The result of this automation has manifested the presence of smarter and intelligent objects, thus paving a way in all spheres: smart cities, healthcare, finance, manufacturing, academia, etc. The application of IoT with percentage implementation in diverse fields is depicted in Figure 1 [1]. IoT is, therefore, an amalgamation of diverse technologies at various layers coming up together to bestow the best of ubiquitous and pervasive computing to provide numerous benefits in different application areas.

Smart services have become an integral part of today’s lifestyle. For example, disabled people could manage things with IoT assistance, specially-abled children could interact using the Autism Glass, and remote health tracking aids in curing. Moreover, IoT sensors working with warning system alerts about environmental disasters. Even the usefulness of IoT in managing natural resources could be realized from the number of use-cases discussed in the literature [2]. With smart grids and smart meters, the daily power-consumption could be optimized and the supply–demand ratio could be efficiently maintained to meet the growing demands. Likewise, intelligent transportation systems provide valuable insights into different services. For example, based on real-time traffic conditions traffic signals consequentially set their timer to avoid traffic congestion and thus, environmental pollution [3]. With smart agriculture, the crop yield could be predicted, fertilizers needed, disease-prone crop areas could be identified and isolated. Alongside these services, it brings deep-rooted security challenges as these IoT nodes are flooded to market with inherent vulnerabilities.

The exponential growth and integration of IoT with other technologies have provided a bigger attack surface to play with [4,5]. Moreover, it is challenging to maintain the security requirements of an IoT system due to the very nature of IoT nodes in terms of scarce resources and unattended environments [6]. Employing existing security mechanisms such as encryption, authentication, and access control is also not a feasible solution for systems with a large no. of connected devices entertaining inherent vulnerabilities. Additionally, the end-users and developers are ignorant about the security risks complimenting the extensive smart applications. These loopholes in IoT devices are exploited to launch cyber-attacks like Mirai [7]. Furthermore, this negligence in securing IoT devices has been proven to be life-threatening. For example, the compromised sensors in self-driving cars could cause human calamity and damage to public properties as well. Now, these cyber-attacks turned out to be another way of declining the economy of the developed countries. Thus, the security challenges being an integral part of these useful IoT services must not be overlooked and should be handled as a priority.

The learning methods are the appropriate tools for differentiating the “usual” and “unusual” behavior of IoT components and the way they interact with each other to provide services. The input to different components of an IoT system is analyzed to find the regular patterns of interaction, to recognize the malicious behavior in a system in the early stages. With learning methods [8] (machine learning and deep learning) nascent zero-day attacks could also be predicted, as these are generally the mutations of foregoing attacks. Moreover, the unique features of deep learning such as automatic feature extraction, compression competencies, etc., make it more feasible for resource-constrained IoT systems. The wide acceptance of deep learning is all due to its ability to self-learning, faster processing, and accuracy. Consequently, IoT systems must have a transition from merely facilitating secure communication amongst devices to security-based intelligence enabled by DL/ML methods for effective and secure systems.

### 1.1. Scope of the Survey

IoT plays a significant role in our lives by enabling the digitization of the physical world around us. A large number of surveys were conducted to review and analyze the multiple IoT facets. Table 1 surmises the relative comparison of the proposed work with the considered state-of-the-art works. However, the study conducted in this paper provides a detailed, in-depth review of those facets/dimensions in an appropriate order. An exhaustive analysis of various research surveys is compiled together to convey an overall assessment, which has not taken place in the past. For example, Neshenko et al. [9] provide a unique taxonomy of numerous attacks and vulnerabilities occurring in IoT devices along with methodologies and security capabilities to counter those flaws. Additionally, architectural vulnerabilities occurring in each respective layer are represented diagrammatically. Furthermore, an appropriate assessment is provided in multiple sections to deliver the essence of the problems occurring due to the coupled nature of IoT devices. Additionally, Butun et al. [10] has shed light on the integration of WSN with IoT and laid stress on the possible attack avenues available generated.

Divyakmika et al. [11] analyzed the application of ML in IoT security by proposing two-tier NIDS. The approach is based on TCP/IP data packet features obtained from NSL-KDD DATASET. It clustered the data into two (normal and new patterns). The classification was done using KNN, MLP, and reinforcement learning. A similar approach is presented by Pajouh et al. [12] to develop an intrusion detection model by collaborating Naïve Bayes and KNN. The challenge of upgrading the mechanism to extend the model to the higher layers is also highlighted. To overcome the problem of availability of the dataset Canedo et al. [13] constructed a testbed to monitor the application of artificial neural networks in attack detection in the IoT sites. However, to generate better analysis, an upgraded testbed with a large number of sensors and devices is required. To construct a real-world attack scenario, Anthi et al. [14] proposed a novel real-time IDS named pulse, which deploys supervised ML for the identification of maleficent activities like scanning, probing, and other elementary forms of DOS attacks with promising results using the Naïve Bayes technique. However, it was executed for the limited number of attacks only. Further, Hasan et al. [15] compared and contrasted the application of multiple ML algorithms in a real-time virtual IoT scenario to further substantiate the research.

Contemporary improvisation includes the application of deep models in IoT. Rahul et al. [16] analyzed the application of various deep models to detect multiple network attacks. KDD cup 99 was used to train the network. However, a lack of real-time IoT datasets and evaluation of deeper networks still posed a challenge. To overcome this, Roopak et al. [17] explored the capabilities of the deeper networks by training models like 1D-CNN, RNN, LSTM, and a hybrid model of CNN + LSTM on the CICIDS2017 dataset. Furthermore, from the considered start-of-the-art, we found that only a few works have explicitly focused on both machine learning and deep learning-based solutions for securing IoT in an elaborated manner. Thus, in this manuscript, we aimed the same. The inherent vulnerabilities in IoT devices and IoT environments (communication protocols) have also been explored as being the root cause of these emerging attacks in smart applications.

**Table 1:** A relative comparison of the proposed work with state-of-the-art works.

Author(s)	Year	Discussion	Challenge(s)	1	2	3	4	5	6	7	8
Ahlmeyer et al. [18]	2016	The different frameworks for securing IoT are discussed and have given their own IoT security framework.	There is no standardization in terms of securing IoT.	✓	✕	✕	✕	✕	✕	✓	✕
Nia et al. [19]	2016	The vulnerabilities in the edge layer of IoT are extensively discussed with mitigation approaches.	The usage of data collected by IoT nodes in unexpected ways.	✓	✕	✓	✓	✕	✕	✓	✕
Alaba et al. [20]	2017	Discussed multiple security scenarios, and possible countermeasures.	To develop lightweight authentication schemes for IoT environments.	✓	✕	✕	✓	✕	✕	✓	✕
Makhdoom et al. [21]	2018	Different malware attacks targeting IoT systems are discussed in an elaborated way.	The challenges IoT will face with FoG computing.	✓	✕	✓	✓	✓	✕	✓	✕
Rahul et al. [16]	2018	Discussed the application of deep models as IDS to detect attacks of varying complexity.	Lack of real-time IoT dataset, evaluation of deeper networks.	✓	✓	✓	✕	✕	✓	✓	✕
Samaila et al. [22]	2018	IoT threat model is given with multiple threat mitigation approaches.	Nano-electronic-based security mechanisms to be explored by IoT.	✓	✕	✓	✓	✕	✕	✓	✕
Butun et al. [10]	2019	Analyzed the application of WSN in IoT. Moreover, an in-depth review of various attacks constituting WSN in IoT.	A better Approach/standard for the routing, trust management, and schemes for data collection for the multiple IoT layers.	✕	✕	✓	✕	✕	✕	✓	✕
Neshenko et al. [9]	2019	Provides a detailed analysis of IoT along with its various facets. Additionally, a taxonomy constituting various attacks, vulnerabilities, and methodologies to monitor them are discussed.	More detailed investigation to provide prompt remediation for detecting malicious IoT devices.	✕	✕	✓	✓	✕	✕	✕	✕
Hasan et al. [15]	2019	Provides a detailed framework for attack and anomaly detection in IoT using machine learning.	More robust algorithms are required; more attention is required for real-time detection.	✓	✓	✓	✕	✓	✕	✓	✕
Roopak et al. [17]	2019	Focussed on the detection of DDoS attacks using deep models along with numerous other challenges in their application.	Lack of Deep learning models that can work with highly unbalanced datasets.	✓	✓	✕	✕	✕	✓	✓	✕
Hussain et al. [6]	2020	IoT security with learning-based solutions is talked over.	The IoT data-based challenges to be explored.	✕	✕	✓	✕	✓	✓	✓	✕
Anand et al. [5]	2020	IoT vulnerabilities and their assessment techniques, with a case study on Sustainable Smart Agriculture.	Lack of intelligent vulnerability assessment technique.	✓	✕	✓	✓	✓	✕	✓	✕
Yazdinejad et al. [23]	2020	Applying blockchain in IoT for secure data transmission and access control.	Comparative analysis with other such architectures.	✕	✕	✓	✓	✕	✕	✓	✕
Rachit et al. [24]	2021	IoT threats, security models, and standardization practices are discussed.	Learning-based solutions will be explored further.	✓	✕	✓	✕	✕	✕	✓	✕
Rasheed et al. [25]	2021	A systematic survey of recent learning-based solutions for securing IoT.	Growing vulnerabilities are not discussed.	✕	✓	✓	✕	✓	✓	✓	✕
The Proposed one	2021	Machine learning and deep learning-based IoT security mechanisms with comparative analysis.	Hybrid learning-based techniques will be explored.	✓	✓	✓	✓	✓	✓	✓	✕

Notes: 1: Architecture; 2: Dataset; 3: Attacks; 4: Vulnerabilities; 5: Machine learning-based IoT; 6: Deep learning-based IoT; 7: Emerging Challenges; 8: Testbed. Notations: ✓: considered; ✕: not considered.

### 1.2. Contributions

The key contributions of this paper are as follows:

- A taxonomy that focuses on attacks, vulnerabilities, and anomalies in IoT is given.
- The benefits of the growing usage of machine learning and deep learning techniques for securing IoT are highlighted. Critical analysis of different learning techniques has also been presented.
- The case studies on the usage of IoT, learning methods, and security challenges in Smart Healthcare System, Smart Vehicular system, and Smart Manufacturing is presented.
- Finally, research challenges and future recommendations for the end-users were given to ensure secure IoT infrastructure.

### 1.3. Methods and Materials

**Figure 2:**
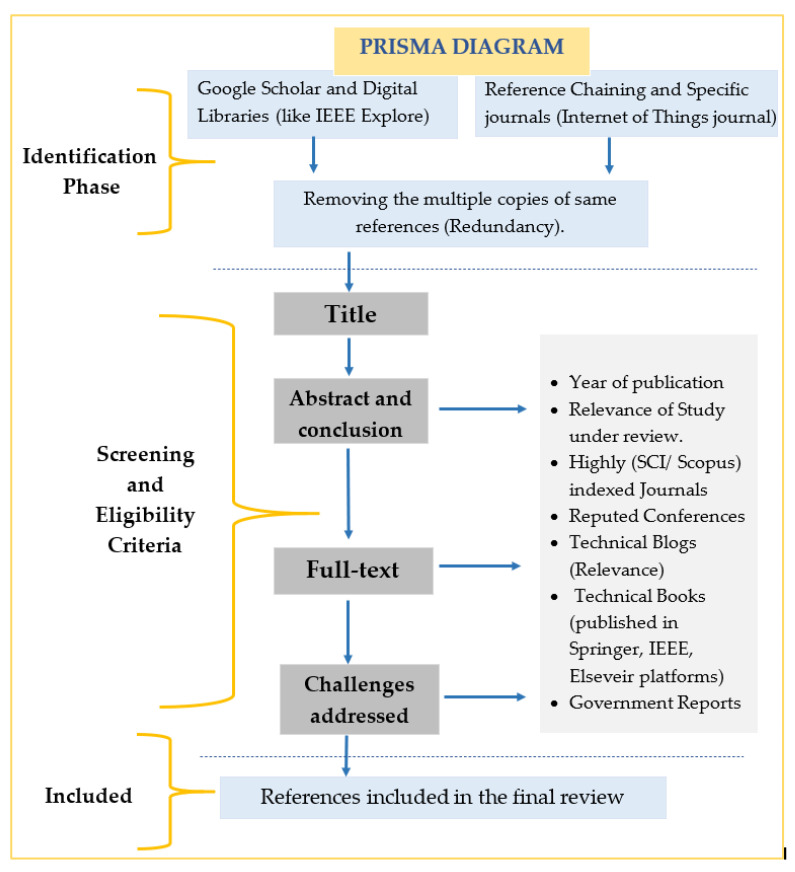
Prisma Diagram of the proposed survey.

The methodical approach is adopted to conduct this study in a proper way to provide in-depth analysis of different learning methods used to secure the IoT system in one way or the other, as security in IoT questions its sustenance. The related research articles, blogs, use-cases, tutorial papers, reports, and white papers were discovered to conduct this review. This work primarily focused on the state-of-the-art research on IoT attacks, threats, anomalies, vulnerabilities, and learning-based approaches to handle them in general and concerning smart healthcare specifically. Additionally, to emphasize the current research challenges, open issues, and future scope related to the same. The screening of the identified articles is done based on the relevance and other factors as depicted in Figure 2. The quality checks are applied to the extracted data to get reliable material for the proposed survey. The ones from the SCI journals and with a good number of citations are commonly chosen. The peer-reviewed and high-quality database journals and reputed conferences like IEEEXplore, Springer, MDPI, Wiley, ACM, Elsevier, and Google Scholar, are investigated to get the relevant research articles. For searching, vital keywords like IoT, security, attacks, vulnerabilities, threats, machine learning, deep learning, smart healthcare, etc., were benefitted.

### 1.4. Organization

**Figure 3:**
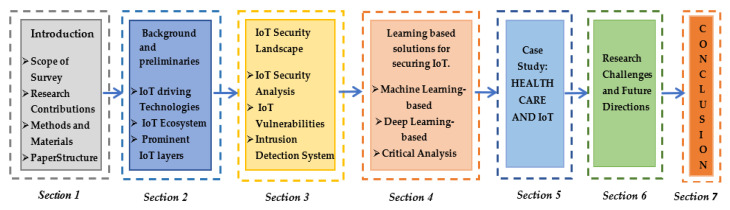
The workflow of the paper.

Figure 3 demonstrates the organization of the proposed article. In Section 1 we present an introduction to IoT and its services, several security issues and attacks, and how ML/DL methods can be the conceivable solution. Section 2 provides a general perspective to the technology and its applications followed by background information, which prominently includes its prime driving technologies, architectural view, and protocol suite. Section 3 introduces security-related concepts by highlighting imminent attacks, anomalies, and vulnerabilities in this area with a brief introduction to the IDS mechanism. The next section presents ML and DL-based IDS solutions to deal with the security intricacies mentioned in the previous section, followed by case studies to understand the practical implementation of IoT in the healthcare sector, vehicular systems, and manufacturing along with research challenges, open issues, and future scope.

## 2. Background and Preliminaries

This section focuses on the background and importance of security in IoT. This section is bifurcated into three subsections. Firstly, we cover IoT driving technologies which include RFID, sensors, wireless sensor networks, communication, cloud computing, and embedded systems. Secondly, we briefly discuss the IoT ecosystem, followed by the IoT architecture with protocol suite in the subsequent subsections.

### 2.1. IoT Driving Technologies

IoT systems consist of various technological/functional components to lubricate the task of sensing, identification, communication, analysis, and management. Colakovic et al. [4] detailed the vision towards IoT along with various technologies used at different levels. Moreover, the survey conducted in [1,5] also introduces these technologies.

- RFID (radio-frequency identification) Technology: It is a technology used for the identification of a person or any other object by exercising the wireless radio frequency technology in the network. It utilizes the labels/tags on the objects for identification. It is a combination of e-labels, an integrated circuit for processing information by modulating and demodulating the signals along with a reader–writer system [26]. Jia et al. [27] presented detailed interpretation and applications of RFID in IoT.
- Sensor Technology: It is responsible for interacting with the physical environment and subsequently detecting, observing, storing, and providing the necessary information by converting it into a human-readable form. The primary purpose is to interpret the real-world conditions by monitoring the documentation collected in the form of sound, light, humidity, pressure, and many other values for analysis of various surrounding scenarios [28]. These, therefore, bridge the gap between the physical and the digital world.
- Wireless Sensor Network Technology: It is an integration of numerous self- configurable devices with embedded sensors for scanning and documenting the conditions of the physical environment and subsequently forwarding them to the appropriate sink node for analysis [29]. Actuators can also be a part of WSN in certain conditions; hence they are often referred to as wireless sensor and actuator networks. The various applications of WSN include weather monitoring systems in which nodes collect temperature, humidity, and other data, soil moisture monitoring system, health monitoring system, etc. For the communication between various sensor technologies, numerous short-distance communication strategies are available like Bluetooth, RFID, Zigbee, Wifi. These are termed network communication technologies. Each one has its pros and cons, and further subsequent selection depends on the application scenario.
- Embedded System Technology: This is a blend of numerous peripheral hardware (Sensors, Actuators) combined with software running or embedded OS (Real-time operating system) to accomplish some specific tasks. Principal components include microcontrollers, memory, network units, ic running on an embedded operating system such as (RTOS) with critical features like real-time computing, low maintenance, and low power consumption [30].
- Cloud Computing: It is an essential IoT component provisioning the users with processing and storage capabilities on demand. It is used as a powerful tool in IoT to handle the big-data and, in turn, rendering intelligent monitoring and decision making in various applications, thus turning them smart. The prime benefits are elasticity, agility with less deployment time [31].

### 2.2. IoT Ecosystem

**Figure 4:**
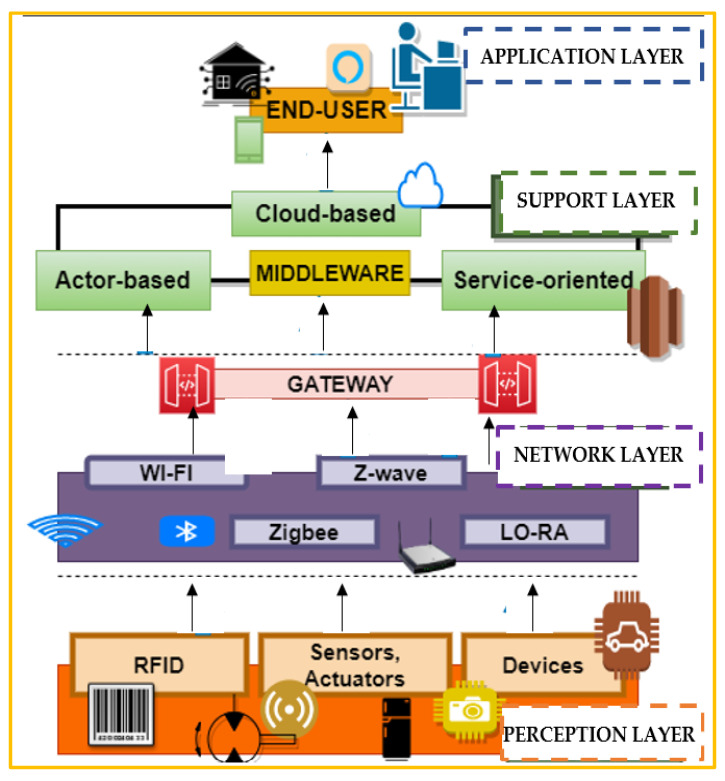
IoT Architecture.

The technologies mentioned above provide a hazy overview of the IoT. To get a crisp and unclouded perspective, understanding IoT architecture is extremely vital before proceeding into the intricate details of the various facets of it. It is hugely challenging to standardize one architecture for IoT due to its inability to capture a particular image characterizing it due to vast expansion and variation in this sector. There are miscellaneous three, four, five, and seven-layer architecture, which are accepted by various professionals to have a visual sculpture of this technology. Table 2 describes some of the prominent IoT architectures. Figure 4 depicts the general three-layer architecture [12,13] with its extension into five layers [14,15].

### 2.3. The Prominent IoT Layers

The two most prevalent architectures IoT-A (internet of things-Architecture) and IIRA (industrial internet reference architecture) synchronized with the IoT community and incorporating multiple views are given in [40]. In concern to IoT, many different wired and wireless protocols are introduced despite the similarity towards the general TCP/IP stack, primarily because of the differences in the characteristics of IoT devices concerning memory and computational power. Priyadarshi et al. [41] and Sahrawi et al. [42] provides a detailed analysis of various IoT protocols. The prominent IoT layers with working protocols are briefly described subsequently.

Perception Layer: It is also referred to as the physical layer in IoT. It is an amalgamation of a wide variety of sensors, actuators, and devices mainly for data accumulation from the surroundings [43]. The primary objective is to acquire all the essential insights for more in-depth analysis in the succeeding. The connected objects should not only establish communication with their respective gateways but also must be able to recognize and talk to each other to merge in real-time to leverage the benefits of the technology. Lightweight M2M (machine to machine) has become a standard for low memory, lightweight devices that typically find an application in IoT [44]. However, such a dynamic approach is disrupted by some of the significant threats by the intruder [3,9,21].

- Node Capture and Cloning: It is one of the most detrimental attacks faced by this layer. In this attack, the intruder gains full control over the IoT nodes. Such IoT nodes can be cloned to launch new attacks.
- Eavesdropping: In this attack, the intruder intercepts the personnel user data. The attacker takes advantage of the insecure communication mode to gain access to such sensitive information.
- Jamming attack: This includes scrambling a particular communication channel by the emission of the radio signals. This attack disrupts the node signals by efficiently bypassing the physical layer protocols.
- Resource depletion attack: This includes multiple retransmissions and collisions of the nodes to deplete it completely.
- Relay attack: This involves the relocation of the IoT nodes with the relay node. The transmitted information passes through the relay node and thus can be exploited by the intruder.

Network Layer: The main goal of the network layer is to establish communication amongst smart devices via the assistance of appropriate IoT protocols. The prime purpose is to transfer data to proper edge infrastructures or cloud-based platforms through intermediaries like gateways or any other data collection systems. Another important aspect here is security. Appropriate security tools like NIDS or any other form of encryption can be applied to reduce the risks of threats and attacks. However, such transmission can be exploited to launch various attacks like a man-in-the-middle attack, routing attack, DDoS attack, Sybil attack.

Support Layer: It consists of cloud-based applications with prime tasks of storing, processing, and analyzing the data. It is mainly referred to as the brain in the IoT body. The main challenges faced here are restricted access and slow data transfer rate, which ultimately leads to late response. These challenges necessitate the need for appropriate edge analytics for quicker replies [44]. DoS and malicious insider attacks are some of the common attacks performed in this layer.

Application Layer: The last layer is the application layer, which ensures data integrity, confidentiality, and authenticity by enabling process-to-process communication via the use of suitable ports. It is responsible for the dispatching of the required services to the end-users via the assistance of appropriate audio and video interfaces. However, several security disputes prevent its proper functioning.

- DoS attack: In this attack, the intruder pretends to be an authenticated user to disrupt the normal functioning of the network. It is accomplished by flooding the authenticated user to trigger a crash [5].
- Phishing attack: It is a sensitive social engineering attack to gain access to the user credentials like passwords, credit card details by masquerading oneself as a trusted body [8].
- Malicious code injection: In this attack, the intruder injects a malicious code to manipulate the authentic data of the authorized user [21,45].
- Session hijacking attack: This attack consists of exploiting the web session by the intruder to gain access to the sensitive data of the user [9].

## 3. IoT Security Landscape

Security is a crucial zone of this technology, as recent trends and surveys have captured numerous changes in this sector, which in turn, indicates the evolution of the attacking mechanism leading to the generation of several zero-day attacks [46]. This behavior is mainly because most vendors are only concerned about dealing with some aspects of the IoT ecosystem. Those involve mostly providing new functionality to get their products into the market and thereby ignoring the privacy and security risks associated, thus making them easy targets of the hackers. The past few years have already recorded some damaging effects of lack of security in IoT in the form of attacks like Mirai botnet attack, Bashlite attack, and many more. Attackers are not only inaugurating numerous scanning, probing, and flooding attacks but are also escalating malware in the form of worms, viruses, and spams to exploit the weaknesses of the existing software, thereby causing severe damage to the sensitive information of the users. Therefore proper detection and prevention of such threats are very vital. IDS provides a platform to deal with such issues. Table 3 and Table 4 provide a brief insight into various such attacks and anomalies at different IoT levels and layers [30,31,32]. Adversaries primarily try to detour the security framework with subsequent launching of zero-day attacks, which in turn reduce the network throughput and produce huge discomforts to the legitimate users [47].

### 3.1. IoT Security Analysis

The listing of various attacks and anomalies prescribes the difficulties in the construction of a secure smart network. The prime goal is to safeguard the security requirements (integrity, confidentiality, availability) of legitimate users. Various researchers have carried out a rigorous survey to list down all possible attacks, their nature, challenges, and countermeasures to deal with them.

Sadique et al. [53] highlighted the critical future security challenges in IoT and open issues w.r.t the various IoT layers. Additionally, Riahi et al. [54] presented a roadmap to IoT security by representing a systemic approach to it by discussing its every aspect, beginning from persons/nodes to the ecosystem to managing privacy, trust, responsibility in the technology via the assistance of a smart manufacturing case study. Mardiana Binti et al. [55] discussed all recent trends in IoT security from 2016 to 2018. Additionally, a layer-wise security approach in IoT with all possible attacks, tools, and simulators is discussed.

Gudymenko et al. [56] present a list of various critical challenges in IoT, required to be addressed to maintain security in this area. Whitter et al. [57] presented a research paper that primarily focuses on the various historical attacks and malevolent activities that happened against the IoT networks. Additionally, the solutions to deal with them and possible areas for future developments are mentioned.

Benzarti et al. [58] presented a taxonomy of attacks against IoT by categorizing them into six classes based on architecture, attributes of security (integrity, authentication, confidentiality), communication disturbance, faulty or corrupted packets, channel, device functionalities. Additionally, the solutions to various existing attacks in different IoT applications like smart grid, smart home, VANET (vehicular ad-hoc networks) are discussed. Additionally, the survey conducted in [44,45,46] provides different IoT attack taxonomies and countermeasures to deal with it.

### 3.2. IoT Vulnerabilities

**Figure 5:**
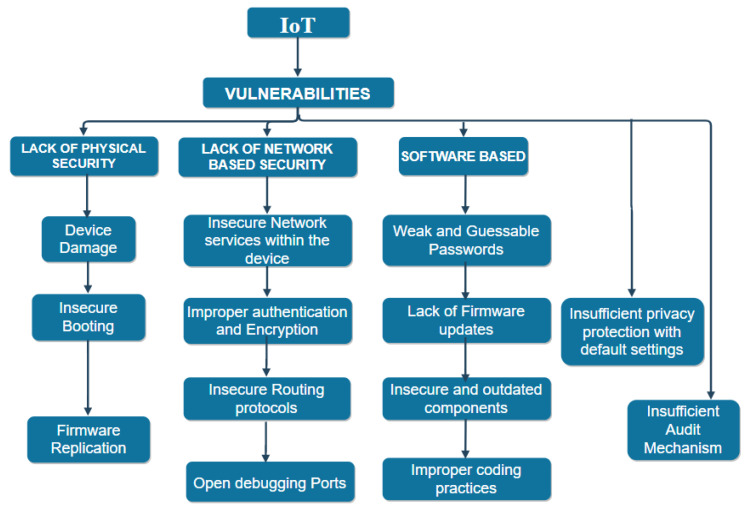
Vulnerabilities in IoT.

Vulnerabilities, in general, refer to the weaknesses of a system that can be overburdened by the adversaries to perform unintended activities. In IoT, hackers can exploit the integrity, confidentiality, availability of services to legitimate users by taking advantage of such teething problems [59]. Therefore an understanding of such delicacy in the system becomes mandatory before the development of appropriate defense mechanisms. The authors presented a multidimensional view of the IoT vulnerabilities with a detailed explanation of their effects on the diverse security paradigms [9]. OWASP (Open web application security project) has also listed the top ten IoT vulnerabilities [60]. Figure 5 explains the prime categorization of various IoT vulnerabilities.

- **Device Security**: This aspect of security surface primarily includes physical damage to the IoT devices mainly caused by unauthorized access to them. The foremost reason is that these devices are in open territory, thus wholly left at the disposal of nature and adversaries. Therefore, they are easily getting damaged, or hackers can clone the firmware to produce their malicious counterpart and can also manipulate the data. Typical examples include the cloning of radio frequency signals in electric cars to unlock them or gaining access to the controller area network bus of the vehicle to execute any damaging activity.
- **Insecure Booting**: Lack of proper verification before the implementation of the device refers to insecure booting. This aspect is an essential requirement in terms of maintaining security because it provides a comfortable surface for attackers to launch their malicious activities by injecting the devices before their launch [61]. The experiment conducted by researchers in [62] against the nest thermostat and Nike + Fuel band, a wearable device to depicts the detrimental effects of the booting process.
- **Network-Based Vulnerabilities**: These typically target the connectivity of IoT devices, thus making them susceptible to a large number of attacks. These typically include the insecure services within the devices themselves, lack of proper authentication and encryption, i.e., using default or weak passwords, and deploying encryption techniques that do not match the standards of lightweight cryptography in IoT, thereby hampering the security. The intruder can perform attacks like DDoS, Sybil attack or could also steal valuable data via the network vulnerabilities. Further due to limited memory and resources in the IoT devices it lacks appropriate encryption to protect the data. In the medical field, attackers can gain control over external devices like insulin pumps or cardiovascular objects to play with the health of people [63].

**Figure 6:**
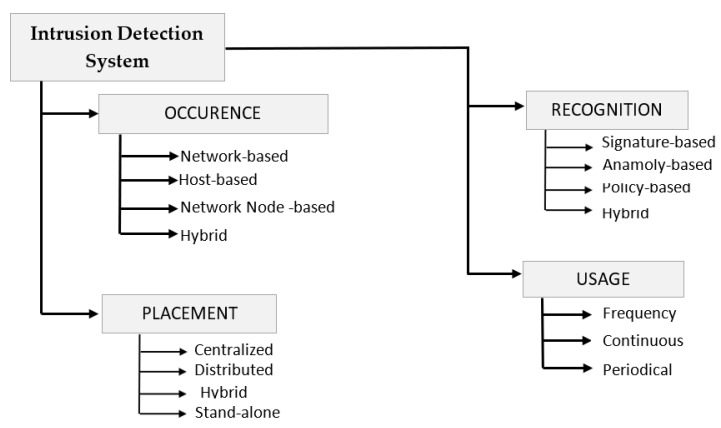
Intrusion Detection System.

Therefore, a deficit of a robust crypto-algorithm makes the devices further vulnerable. Research work related to authentication and encryption is provided in [64,65], respectively. The situation is further worsened via the presence of open ports. These are a significant threat to the IoT devices because they can expose the existence of smart devices in the surroundings, thus providing a platform to adversaries to conduct mischievous activities like modification of the firmware, injecting malicious code. The well-known Mirai botnet attack took the advantage of the open telnet ports to create an army of multiple compromised devices on the internet. To further fulfill its intentions Mirai used a brute force approach by attempting default factory credentials or the dictionary of attacks to generate the username and the password. Sivanathan et al. [66] explained the use of SYN and TCP scans to discover IoT devices at the disposal of open ports. Further, Markowsky et al. [67] described the usage of dark web SHODAN [68], Masscan, and NMAP to find and connect to vulnerable devices in the network.

d. **Software-Based Vulnerabilities:** These typically include the usage of readily available, guessable, and default passwords, also in addition to this, not performing suitable software updates/patch updates or using deprecated or outdated software libraries or components. All these factors together increase the vulnerability of the entire system [69] explains the attacks launched due to firmware modification. Further, deliberately following weak programming practices, i.e., launching firmware with well-known vulnerabilities, aids hackers to perform their dark activities.
e. **Insufficient Privacy:** This means compromising user’s personnel information without seeking their permission because of current default settings that often restrict users from altering the configurations. This can be life-threatening in the case of e-health services. A pacemaker with wireless capabilities was found vulnerable thereby exploiting the health of the user [70].
f. **Insufficient Audit Mechanism:** Lack of sufficient logging mechanism lead to such vulnerabilities. The research survey in [60,61] provides some insights towards audit mechanisms in IoT. Figure 6 depicts the most vulnerable IoT devices by 2020. The devices, mainly security cameras, virtual assistants, smart TVs, and smart lights, have proved to be the most vulnerable towards adversaries [71]. These devices can be easily hijacked to perform both active and passive attacks. In the case of security cameras, mainly, the fault lies at the purchase corner of these. Buying cheap models can open doors for hackers. Similarly, in the case of home assistants, eavesdropping may be a carrier of your activities to the adversary. Additionally, remote access to various devices can be undertaken to perform all kinds of mischief [72].

### 3.3. Intrusion Detection System

Several countermeasures are proposed to deal with the wide variety of attack scenarios in IoT. These vary from better authentication, device identification to introducing lightweight encryption to several others like adding risk assessment models, and intrusion detection at higher layers of IoT. In this survey, we particularly narrowed our research to IDS-based attack and anomaly detection. It is defined as an appropriate ensemble of various tools, techniques, and methods required to detect unintentional activities of the hackers.

Figure 6 provides a view of the multiple properties of IDS like its occurrence, placement, recognition strategy, and usage frequency, the knowledge of which is essential for its proper implementation to achieve the desired results. The properties are described in terms of whether they are host-based or network-based, i.e., deals with attacks and anomalies launched against the entire network by analyzing all the incoming packets in the system. Snort, Suricata, Zeek are some of the examples of NIDS, or they can be hybrid, i.e., composed of both HIDS and NIDS. It is referred to as the network monitoring stage of IDS, which is followed by analysis. Finally, the detection stage, which is again categorized into misuse-based, anomaly-based, or can be policy-based [63,64]. There are several IDS techniques based on data mining, ML, statistical model, payload model, rule-based, but due to the massive data generation in IoT, ML can be thought of as a suitable paradigm to provide intelligence in this area. It can leverage the vast data generated by IoT devices for training to create patterns and behavior to make appropriate predictions and assessments. Thus IDS based on ML-based learning approaches can prove to be an excellent tool for attack detection in a smart IoT environment.

## 4. Learning-Based Solutions for Securing IoT

**Figure 7:**
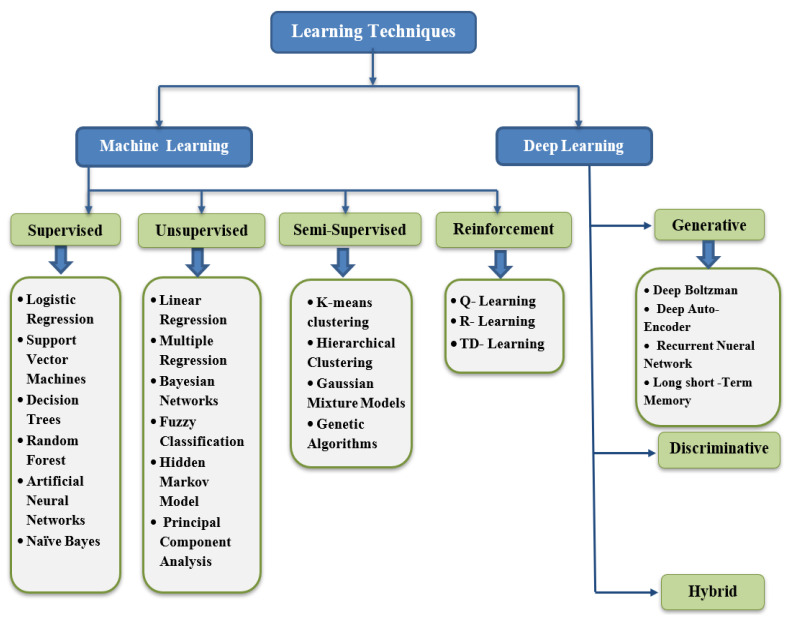
Various Learning approaches.

**Figure 8:**
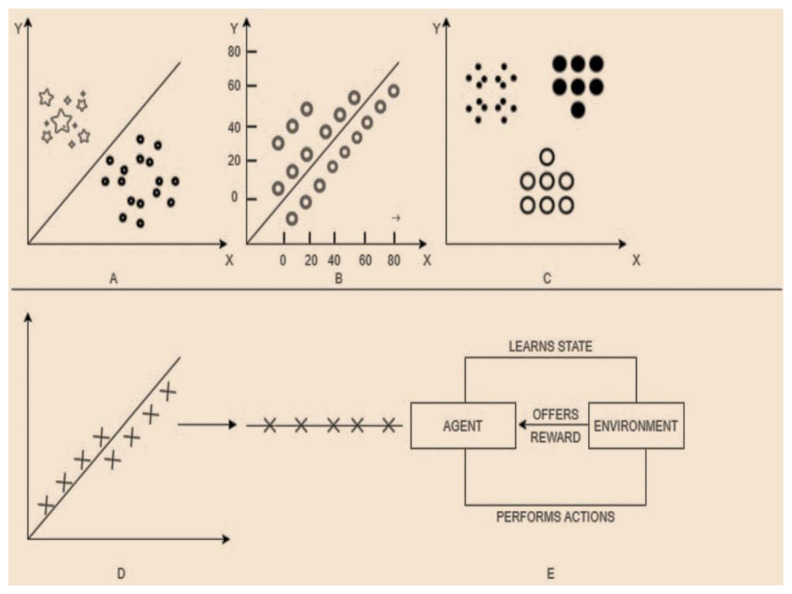
(**A**) Classification. (**B**) Regression. (**C**) Clustering. (**D**) Dimensionality reduction. (**E**) Reinforcement.

The vulnerabilities, attacks, and anomalies mentioned in the previous section focused on the broad range of concerns brought in our lives due to the expansion of IoT. Additionally, the advances in big data and computing power have further surfaced the platform for carrying out unintentional activities by the adversaries. However, ML-based specialists identify learning approaches as a productive tool to deal with IoT-based security issues, thereby leading to the amalgamation of ML and DL approaches with IDS technology. Figure 7 depicts a classification of existing learning techniques. In this section, we will mainly focus on various learning approaches, their types, and multiple solutions for IoT security based on these approaches. Existing methods can be classified based on the mode and the approach used. Figure 8 provides a visual sculpture of these.

- **Based on the mode:** There are two modes: offline and online. In offline mode, the input is processed in batches and is known as lambda learning, whereas in online mode, the data are processed piece by piece serially and is known as kappa learning.
- **Based on the approach:** There are three approaches: supervised, unsupervised, and reinforcement.

**Supervised Learning:** It is a procedure of learning the functionality from the training dataset. The prime goal is the estimation of the mapping function to predict the correct output labels for the prescribed new data. Based on the essence of target labels, it can be classified into classification and regression [73]. The technique is enormously useful in fault detection and misuse-based intrusion detection, quality of service, event detection, etc. The prime prerequisite in implementing supervised ML algorithms in IoT is the availability of the dataset with signatures for known attacks for learning purposes. There are various supervised learning approaches like Knn [74], Decision tree [75], SVM [76], Naïve Bayes [77], ANN [78] utilized for attack detection in IoT. Despite high detection statistics, lack of detection of different attack footprints, more resource consumption limits their usage in the era of numerous Zero-Day attacks.

**Unsupervised Learning:** It is very useful in modeling the elementary or the concealed structure of the data due to the non-availability of the labeled dataset. The unavailability of the labeled dataset differentiates it from the supervised approach, thus promotes a comprehensive evaluation of the data. It is majorly bifurcated into three sections, namely clustering [79], dimensionality reduction [80], and density estimation. Hence, these approaches are instrumental in detecting outliers and novel anomalies. Additionally, Dimensionality reduction techniques like PCA helps in eliminating the features which have no contribution to class separability.

**Reinforcement Learning:** The technique is concerned with the application of appropriate actions taken by the software agents in an environment to maximize the cumulative reward. More generally, it can be a catchphrase as learning from the environment. Two principal methods of reinforcement learning include policy search and value function approximation. The primary classification includes Q-learning, TD-learning, and R-learning. The mentioned ML classification techniques with their pros and cons indicate that there is no particular algorithm that is applicable in all the situations. Additionally, the increase in the number of IoT devices and the continuous evolution of zero-day attacks have urged the researchers to come up with Ensemble, hybrid, and other fused models to overcome the pros and cons of individual classifiers. Figure 8 depicts various learning models of machine learning.

**Federated learning (FL):** Another thriving machine learning paradigm that is capable of sorting the issues of security in IoT devices is federated learning (FL). This advanced machine learning technique is capable of training the machine learning models in a distributed manner. Traditionally, there was a significant communication overhead during the transmission of updates between the centrally managed server and the connected devices in the network. The network overhead leads to compromise the data rates, reliability, privacy, and resource management [81]. However, with the advent of FL methods, there is a significant improvement in the security aspect of smart systems. The learning models under FL takes the advantage of the distributed nature of learning and ensure the transmission of only learnable parameters instead of whole datasets. FL has been of immense use in intelligent transport systems thereby ensuring the security and privacy of data.

### 4.1. ML-Based Solutions for IoT Security

Arthur Samuel coined the term “Machine Learning“ in 1959 and defined it as a field of study that gives computers the ability to learn without being explicitly programmed [67]. It is used to comprehend a model defining the particular behavior or characteristic and then subsequently utilizing it to predict the traits in seen or unseen instances. The flexibility, adaptability, and low CPU load of ML algorithms can help us build numerous analytical models with better accuracy and reduced false alarm rates for attack and anomaly detection. Further, understanding various ML approaches is a prerequisite to understanding their suitability towards various attacks and anomalies. Table 5 summarizes the different machine learning-based solutions to secure IoT systems against the growing attacks.

Anthi et al. [14] proposed novel real-time IDS named pulse, which deploys supervised ML for the identification of maleficent activities like scanning, probing, and other elementary forms of DOS attacks. In this work, the authors developed a smart home testbed and with cross-validation concluded the better results by using the Naïve Bayes technique. In a similar work [11], a two-tier machine learning-based NIDS is proposed with preprocessing using wekas and the construction of an autonomous model based on hierarchical agglomerative clustering. Additionally, Pajouh et al. [12] introduced a state-of-the-art technique for subsequent detection and classification of malignant activities like the user to root and remote to local attacks by acquainting the readers with TDTC (two-layer dimension reduction and two-tier classification module) model. Both PCA and LDA are employed to reduce the computational complexity, then succeeding forward by the application of Naïve Bayes and CF-KNN along with the KD tree to present a more efficient classification.

Shahid et al. [82] presented a smart home monitoring system to generate legitimate traffic data with the malicious traffic created offline by deliberately attacking the device or by using IoT honey-pots. Six machine learning algorithms were deployed, followed by a comparison of their accuracies in which Random Forest outperformed. In another work, Srinivasan et al. [83] leveraged the power of machine learning techniques like random forest, support vector machine, MLP (multilayer perceptron) to ease the recognition and localization of link faults in the highly sophisticated network like IoT using a mininet platform.

Moustafa et al. [84] proposed an Adaboost ensemble model (Decision tree, Naïve Bayes, ANN) to detect malevolent activities, particularly attacks in the network by using features of DNS, HTTP protocols in TCP/IP models. It is a three-step framework initialized by feature extraction by using Tcpdump, Bro-ids, and other extractor modules followed by generation of data-sources from UNSW-NB15 and NIFS dataset and simulated IoT traffic. In [13], the authors conducted suitable experimentation to generate their own synthetic data to inspect and carefully scrutinize the usage of ANN (Artificial Neural Networks) in IoT gateway devices present in the transport layer to work at the security aspects of the technique. Further, Ioannou et al. [85] presented an ML approach known as a support vector machine for the detection of malicious activities within the IoT network exploiting actual IoT traffic with specific network layer attacks such as blackhole, selective forward, etc.

On similar lines, Zhao et al. [86] proposed a novel framework for real-time intrusion detection for numerous attacks and other suspicious activities occurring at the network layer using online machine learning with better time complexity using softmax regression. In [87] the authors presented an online sequential extreme learning machine model for intelligent detection of attacks at the fog nodes to provide a faster, scalable, and flexible interpretation of benign and adversarial traffic coming from the IoT application. In another notable work, Hasan et al. [15] compared the anomaly detection mechanism of various ML techniques (LR, SVM, DT, RF ANN) in a virtual environment producing synthetic data in which random forest outperformed with 99.4% accuracy.

Lee et al. [88] come up with profiling of abnormal activities of IoT devices via the support of a variety of machine learning algorithms. The approach considers signal injection as a threat to IoT and hence finds it as a principal attack in his research. In [89] the authors proposed a unique human in the cycle intrusion detection via ML to reduce the dependency on a large amount of labeled data for anomaly detection exploiting the query selection mechanism for unlabelled data. Further, Shafi et al. [90] presented a fog-aided SDN (software-defined networking) structure for anomaly detection and prevention for IoT networks, mainly to overcome the pitfalls of screening at the cloud and at the devices, evaluated by simulating an IoT network using the cooja simulation tool. However, due to certain limitations like processing power, scalability, manual feature selection, and heterogeneous data handling pushes us to come with better learning approaches. To deal with some aspects of limitations in ML, DL was implemented and analyzed in the security region of IoT [91].

### 4.2. Deep Learning-Based Solutions in IoT Security

Deep learning technology is considered to be a successor of ML with the capability of mimicking the human brain, thus falling under the categorization of AI. Deep networks have the potential of achieving better accuracy in terms of predictions and classifications because of the multilayered composition. This composition, when combined with IDS, can achieve performance at a superhuman level for the detection of new attacks and anomalies [16]. The principle benefit of the technology is the omission of manual feature selection and the capability to model non-linear relationships, thereby achieving an edge over ML. Moreover, the ability to handle Big Data, automatic feature extraction further backs the usage of technology in IoT. The essence of the technology revolves around cascading multiple layers for predicting the output. To accomplish the non-linearity activation function plays an important role. Table 6 lists the activation function for deeper networks [92]. Furthermore, Table 7 summarizes the different deep learning-based solutions used to secure IoT systems. Deep learning can be classified into three classes, known as discriminative, generative, and hybrid models.

**Discriminative Models**: These models belong to the class of supervised learning and thus are used for treating problems of classification and regression. If the input label is X and the corresponding output label is Y, then discriminative models require to learn the conditional probability of target label y, i.e., p(y|x) [93].

**Convolutional Neural Network (CNN):** It is a feed-forward deep artificial neural network that leverages the concept of convolution for predictions. The notion is to allocate importance to different parts of the image by connecting only a smaller region of a particular layer to the layer, succeeding it. The primary concept is to reduce the size of weights and the neurons. The functionality of CNN revolves around the four layers, namely the convolution layer, to reduce the size of weights followed by the Relu layer to introduce non-linearity into the network [94]. Then come the pooling and the fully connected layer, which subsequently perform the task of shrinking the stack size obtained from the previous layer and performing the actual classification, respectively. Nowadays, the technique is finding usage in the sector of anomaly detection [93,94], the approach is fused with other methods for anomaly detection, thus providing a profitable proposal in this sector.

**Recurrent Neural Network (RNN):** This type of feed-forward artificial neural network posses internal memory. The associations between the various units form a digraph, thereby allowing the structure to copy the output and propagating it back to RNN at every timestamp. These associations permit the composition to evince temporal dynamic behavior. The characteristics mentioned above make it appropriate for applications like speech recognition, time series prediction, and anomaly detection [95]. There are many variants to the basic RNN, namely hope field network, fully recurrent, Elman and Jordan networks, etc.

**Long Short Term Memory (LSTM):** It is a type of RNN with an ability to remember long-time dependencies, thus overcoming the limitations of RNN. The composition of LSTM includes memory cells for keeping back the information along with three gates, namely forget, input, and output for memory orchestration [96,97].

**Generative Models**: These models belong to the class of unsupervised learning. They are used when there is no presence of labeled data. The model requires calculating the joint probability p(x,y) where x and y are input and output variables, respectively.

**Table 5:** Tabular Representation of Machine Learning Approaches.

Author	Algorithm with Implementation Platform	Threats	Challenges	Performance Evaluation
Anthi et al. [14]	Naïve-Bayes Platform: Weka	Network probing, scanning, Dos attacks-SYN, UDP flood attacks.	No clustering of similar devices, limited attacks covered.	scan attack: precision-97.7, recall-97.7, f-measure-97.7 SYN: precision-80.8, recall-68.8, f-measure-65.8
Divyatmika et al. [11]	Clustering+ KNN(data classification) + MLP (misuse detection) + reinforcement(anomaly detetion) Platform: Weka	Dos, probe, Remote-to-local(R2L), User-To-Root(U2R).	-	Accuracy: 99.95%(with reduced false alarms).
Pajouh et al. [12]	PCA + LDA (Feature selection),naïve bayes + CF-KNN (classification)	Dos, probe, Remote-to-local(R2L), User-To-Root(U2R)	Anomaly and intrusion detection at the application and support layer, considering different protocols of the network layer.	Accuracy: Probe Attack: 87.32, Dos Attack: 88.20, U2R-70.15, R2L-42 Detection rate: 84.86, False alarm rate-4.86
Shahid et al. [82]	Random forest, Decision tree, ANN, KNN, GNB (Gaussian Naïve Bayes)	-	Integration of anomaly detection models with a software-defined networking environment.	Accuracy: RF-99.9%, DT-99.5%, SVM-99.3%, KNN-98.9%, ANN-98.6%, GNB-91.6%
Srinivasan et al. [83]	Random forest, MLP, SVM Platform: mininet	Link fault identification.	Testing different ML algorithms.	Accuracy: 97%
[97]	Ensemble model (Decision tree + Naïve Bayes + ANN) Platforms and tools: NodeRed middleware, tcpdump, Bro-IDS,	Analysis, backdoor, dos, exploit, fuzzers, generic, Reconnaissance, worms.	Considering other IoT protocols, concentrating on ore zero-day attacks.	Accuracy with DNS data source: 99.54%, Accuracy with HTTP data source: 98.97%
Canedo et al. [13]	ANN Platform: R(neural-net package).	Invalid data entries.	Generating data entries by creating a testbed with more devices and sensors.	N/A
Ioannou et al. [85]	c-SVM platform: RMT tool(Run time monitoring tool).	Routing layer attacks (sinkhole, blackhole, selective forward).	Placement of IDS in high-energy gateway nodes.	Accuracy: 100% (with the same topology) Accuracy = 81%(when the topology is changed)
Zhao et al. [86]	PCA (to reduce dimensions) + KNN (classification + Softmax regression (classification).	Dos, probe, Remote-to-local (R2L), User-To-Root (U2R)		Accuracy: 85.24% with 3 dimensions, 85.19% with 6 dimensions 84.406% with 10 dimensions.
Prabavathy et al. [87]	OS-ELM (online sequential extreme machine learning) Platform: MATLAB (R2013a).	Dos, probe, Remote-to-local (R2L), User-To-Root (U2R).	More depth analysis of zero-day attacks is required.	Accuracy: 97.16% (forbinary classification) TPR (true positive rate): normal-98.63%, probe-84.2%, Dos-96.61%, U2R-53.81,R2L-71.87% (for multi class classification).
Hasan et al. [15]	LR, SVM, ANN, RF, DT Platform: python with Numpy, pandas, sci-kit learn.	Dos, data type probing, malicious control, malicious control, malicious operation, scan, spying, wrong setup.	More robust algorithms are required, more attention is required for real-time detection.	Accuracy: LR-98.3% SVM-98.2% DT-99.4% RF-99.4% ANN-99.4%

**Table 6:** Activation Functions.

Activation Function	Nature	Range	Classification	Mathematical Notation	Usage
Sigmoid	Non-linear	0 or 1	Binary classification	f(x) = 1/1 + e^−x^	Output layer
Tanh	Non-linear	−1 or 1	Binary classification	Tanh(x) = 2 × sigmoid(2x) − 1	Output layer
Relu [98]	Non-linear	[0,inf]	Multiple classification	f(x) = max(0, max)	Hidden layer
Swish	Non-linear	-inf to inf	Multiple classification	f(x) = x × sigmoid(x)	Hidden layer

**Autoencoders:** It is a class of deep learning model which relies on the concept of rebuilding the input after performing suitable compression via the application of an encoder followed by a decoder [99]. The prime task is to achieve dimensionality reduction to visualize the data and gather suitable projections from it provided input features are not independent and have some correlation. Vanilla, convolutional, multilayer, regularized are some variants of autoencoders. Meidan et al. [100] presented N-Balot (network-based detection of IoT botnet attacks using deep autoencoders) to detect botnet attacks using autoencoders.

Roopak et al. [17] presented a deep learning-based hybrid approach particularly for DDOS attack detection and comparisons were made with the standalone Machine learning techniques. In another work, McDermatt et al. [101] provided a novel bidirectional long short-term memory-based RNN for the sensing of botnet activities amongst the consumer IoT device. Packet level detection was performed along with word embedding for recognition of text and conversion of packets into integer format. Further, Rahul et al. [16] proposed a deep neural network-based approach to predict attacks on a NIDS.

On similar lines, Diro et al. [102] presented a deep learning model for the distributed detection of attacks to leverage the self-teaching and compression capabilities of DL to implement the network detection of attacks at fog nodes. The results showed that distributed attack detection provided better accuracy compared to the centralized schemes. Further, an attempt to collaborate DL technology with its shallow counterpart was made by Shone et al. [103]. They presented a novel unsupervised learning approach named NDAE (non-symmetric deep autoencoder) for feature engineering combined with random forest for classification.

Ullah et al. [104] proposed a tensor-flow-based Deep neural network approach to detect software piracy and other malware-based attacks in the industrial IoT network. This DNN is used for capturing pirated software from the source code of different programmers from google code jam followed by an application of CNN to detect footprints via binary visualization on colored images of malware files. Traffic classification plays a very vital role in ensuring security in IoT networks. Yao et al. [105] present an end-to-end deep learning-based capsule network approach for traffic classification and identification of malware, unlike the conventional DL methods.

In another work, Telikani et al. [106] proposed a CSSAE technique for intrusion detection, especially in IoT networks. The main focus of the paper is the class imbalance problem in the datasets, which tends to bias the results towards the majority class. Pajouh et al. [107] also deployed LSTM for malware detection in ARM rooted IoT applications. In [108] the authors exploited RNN, and network coding in amalgamation to prevent eavesdropping attacks in heterogeneous IoT environments with highly unreliable storage structures and proposed two algorithms FAGA() (failure-aware greedy allocation) and FLAGA() (failure-and-load aware greedy allocation) to test the failure condition of storage devices.

**Table 7:** Tabular Representation of Deep Learning Approaches.

Author	Dataset Used	Algorithm with Implementation Platform	Threats	Challenges	Performance Evaluation
Roopak et al. [17]	CICIDS2017	MLP,1-d CNN,LSTM, CNN + LSTM Platform: Keras–Tensorflow, machine learning implementation MATLAB2017a.	DDOS	Lack of Deep learning models that can work with highly unbalanced datasets.	Accuracy: 1dCNN-95.14%, MLP-86.34%,LSTM-96.24%, CNN + LSTM-97.16%.
McDermatt et al. [101]	Dataset generated by creating a testbed.	BLSTM	Mirai(scan, infect, control, and attack), UDP.	Lack of comprehensive dataset including more attack vectors.	Accuracy: 99.99% (Mirai), 98.58% (UDP).
Rahul et al. [16]	KDD cup 99	DNN with three layers Platform: Keras (Tensorflow).	Dos, probe, User-To-Root (U2R), Remote-to-local (R2L).	Lack of real-time IoT dataset, evaluation of deeper networks.	Accuracy: 93%.
Diro et al. [102]	NSL-KDD	Deep learning model with 150, 120, 50 neurons in first, second, and third layer respectively.		Implementation of technique on different datasets.	Accuracy: 96% to 99% 99% (for two class-normal and anomalous) 98.27% (for 4 class(normal, dos, probe, U2R and R2L)
Shone et al. [103]	KDD cup 99, NSL-KDD	NDAE (non-symmetric deep auto-encoders) Platform: GPU enabled tensor-flow.	Dos, probe, User-To-Root (U2R), Remote-to-local (R2L	Lack of real-time traffic for appropriate analysis.	Accuracy: 94.58% (Dos), 94.67% (probe), 3.82% (R2L), 2.70% (U2R).
Ullah et al. [104]	Google code jam, Leopard Mobile dataset1	Deep neural networks Platform: Tensor-flow	Pirated software and malware threats(industrial IoT).	-	Accuracy: 96%
Yao et al. [105]	UTSC-2016	Capsuleapproach(1-D CNN + capsule networklayer + LSTM + output layer. Platform:Python2.7, TensorFlow1.8.0	Malware threats.	-	Higher classification accuracy compared to traditional approaches.

The complete inspection and scrutinization of the prevailing ML and DL techniques concerning the survey conducted in this groundwork stipulate the following trends for anomaly detection in the IoT. As a matter of fact, concerning the non-availability of a particular IoT dataset has advocated researchers to orchestrate their experiments either by using some non-IoT series of data or come up with their data records [108,109]. Further, the survey conducted also helps us to reach some conclusions for the learning approaches which includes their advantages, disadvantages, and their suitability towards the various known attacks which is depicted in Table 8.

The table mentioned above will assist readers with the choice of learning approach they want to implement in their researches based on their advantages, disadvantages, and their suitability towards the various attacks.

## 5. Case Studies

### 5.1. Healthcare and IoT

The innovation in numerous IoT technologies has led to the decentralization of healthcare mechanisms from being traditional to a customary localized forum via the assistance of IoT-authorized gadgets. These gadgets are based on the concept of a multisensor framework for recording various parameters. These include recording blood sugar, ECG (electrocardiogram), pulse, temperature, etc. of the patient. This customization supports the notion of remote health tracking, which in particular involves at-home medication, elderly care, or any fitness program [117,118,119]. Healthcare in IoT primarily involves four basic entities, which are actors, sensors, communication networks, and applications. The actors include the patients, clinical staff involving the doctors, nurses, experts. Sensors are used for illuminating the actors with paramount requirements and subsequently dispatching the information via a suitable communication network [120]. There are profuse devices prevalent for reading and tracking vital patient data and other medical statistics. These devices range from smart wearables like smart bands, watches, shoes to intelligent video cameras and meters. Applications assist with real-time notifications, thus aiding any emergency services.

**Figure 9:**
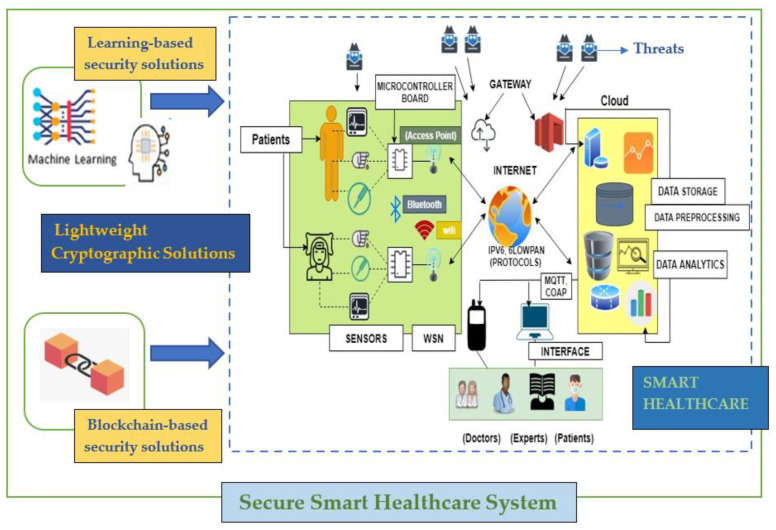
Secure Smart Healthcare System.

The real-time monitoring of data generated by smart devices and their transmission in the ecosystem is very critical to intelligent decision-making. These intelligent systems work autonomously without human intervention and decision regarding mitigating a specific threat is taken in real-time after adapting to environmental changes. Figure 9 depicts secure smart healthcare management with the use of technologies like artificial intelligence, blockchain, machine learning, and deep learning providing autonomous working and decision making. Sensors are used for reading patient’s data and are connected to the microprocessors. These microprocessors are further connected to any wireless communication technology for routing and forwarding the data through the gateway. The data are stored in the virtual machines popular as clouds for preprocessing and analysis. These data can be accessed by doctors, experts, and even patients. However, a proper security mechanism is required to prevent any kind of damage by the adversaries.

Various IoT architectures have progressed over the past years. Some of the prominent architectures are given. For example, mHealth is a primary health care system with a three-layered structure. The layers include a data collection layer for apprehending and collecting the data followed by a data storage layer, which provides for stocking the data in the stack pile racks, and a data processing layer for a proper inspection and scanning of data [121]. Additionally, 6Lowpan consists of numerous access points with forwarding and routing capabilities. The deployed sensor nodes, along with the access points, lead to the formation of clusters. The connection is achieved via the assistance of IPV6. This approach is preferred over others due to its low energy requirements, which makes it suitable for the battery-powered sensor. Gao et al. [122] discuss a Zigbee-based structural health monitoring system. The revolution in WSN allows multiple sensor nodes to communicate wirelessly with the base station. To increase the lifetime of the network, a low-energy communication channel is necessary. This led to the injection of Zigbee for communication in the health monitoring system.

Despite many benefits, this sector of technology suffers from various loopholes, which are enumerated below. The massive growth in the deadly underlying medical conditions of the population requires well-organized, systematic, and efficient healthcare management. Despite the numerous benefits like better diagnosis, treatment, and other facilities, the smart and ubiquitous nature exposes it to multiple cyber threats. Cybersecurity in healthcare is at a nascent stage and thereby requires proactive and improved technologies to protect it from various attacks. Understanding different security challenges are necessary before dealing with other intricacies of it. There are numerous challenges and issues for contemporary health care applications. The broadcast nature of communication in healthcare leads to the exploitation of the privacy of the patients, thus launching platforms for serious threats like eavesdropping. This aspect, in turn, leads to the exploitation of the confidentiality of the data [123]. Furthermore, any change in the data received from the sensors can be life-threatening in the case of healthcare applications. Therefore, integrity and authentication are the two major concerns here. Moreover, the author in [124] depicts how emergency services can be disrupted and compromised because of a lack of a single cloud-based infrastructure where all e-health records can be accessed. Further security breaches in cloud storage can worsen the situation.

To address the above-mentioned flaws, better and improved security frameworks are required that necessitate the amalgamation of machine learning in this sector. Besides fixing critical medical conditions like the identification of tumors, bleeds, etc., this AI tool can solve many security-related affairs and issues by acting as an anomaly detector. Newaz et al. [125] suggested the application of health guard: an ML-based security application framework for healthcare systems. This framework leveraged multiple ML algorithms (KNN, Random Forest, DT, ANN) for detecting malicious activity and was able to achieve an accuracy of 91%. The framework can encapsulate and observe correlations amongst multiple body functionalities and other crucial signs. The structure was tested against threats that included tamped medical devices, DOS, and other false data. To further increase security, research is being carried out to combine ML with blockchain technology.

Tanwar et al. [126] suggested the use of ML in blockchain to improvise data security and privacy. The architecture was proposed by integrating the blockchain with ML. The learning potential of ML combined with blockchain technology that will not only make it smarter but also reduce many data-oriented issues in IoT could be seen in recent works [127,128,129,130]. Decentralization, transparency, and immutability are the primary objectives of blockchain technology, which help to improve the security of the system [131,132,133]. This combination will result in correct predictions and better security. Additionally, Nilima et al. [134] further backed that the usage of ML with blockchain to make the system smarter and deal with privacy, integrity, and authentication issues.

### 5.2. Smart Vehicular System

In addition to ensuring security in the healthcare sector using IoT, there are many scenarios where the application of the internet of things is being realized. Recently, the application of IoT in vehicular security systems has gained huge success and attention [135]. The progression in intelligent technologies has opened a wide array of opportunities for the ever-vulnerable smart vehicle systems. The availability of 4G LTE and 5G communication spectrum has unlocked many possibilities for cyber-attacks leading to compromise of security in smart vehicular systems (SVS) [136]. These connected vehicles are the source of generation to the enormous amount of data and therefore are vulnerable to many security attacks. Some of the popular security attacks on the SVS are Denial of Service(DoS), Blackhole, Replay, Sybil, Impersonation, Malware, Falsified information, and timing attack [137]. All these cyber-attacks attempt to destabilize the functioning and performance of the SVS. The application of intelligence on monitoring and controlling these sensors enabled smart vehicular systems to have made these systems more robust and secure. Deep learning techniques and machine learning-based algorithms like k-NN, SVM, decision trees, etc. are in use for developing a security solution in vehicular systems using IoT. An example of the Tyre Pressure Monitoring System (TPMS) [138] in the intelligent and connected system of vehicles ensures proper monitoring of tyre pressure in all the tyres of the vehicle including the spare wheel in the boot. The system was devised for ride comfort and robust handling of the vehicle on the road. The use of sensors for all the tyres ensures the collection of real-time data for the proper safety of the vehicle. A cyber-attack on this system may leak the collected data to the attacker, thereby compromising the valuable data such as the location of the vehicle, speed of the vehicle, and the braking behavior of the driver [139]. The prevention against such types of attacks using learning-based mechanisms has made this system more applicable in current scenarios [140].

The security challenges the smart vehicles face today could be realized from the severity of security incidents in smart vehicles [141,142]. The infotainment system vulnerabilities are being exploited to get into smart cars [143]. Tesla motors faced the causality in the smart car accident all due to the compromised sensors [144]. Anand et al. [3] discussed the use-case of smart transportation covering the common attack surfaces and inherent vulnerabilities.

### 5.3. Smart Manufacturing System

With the amalgamation of hardware, software, and the internet with IoT, another promising domain with immense potential to improve the global economy is smart manufacturing. The four vital components of any manufacturing unit or organization are processes, people, products, and infrastructure [145]. The application of sensors in any of these four components results in yielding an enormous amount of data which would be very critical for the overall monitoring and control of the manufacturing systems. The main advantage of having IoT in manufacturing is the optimum functioning of these four components. With the benefits of IoT in manufacturing, there are pitfalls too. These smart-systems are vulnerable to cyber threats leading to malfunction of the overall systems [146,147]. However, the ever-evolving use of machine learning and deep learning techniques in manufacturing helps to prevent and mitigate cyber threats. One of the security issues in manufacturing units is the prediction and management of vulnerabilities [148]. In these categories of security issues, the machine learning algorithms are applied to gather the data to identify the areas of the fault occurrences, i.e., to predict future issues from past issues [149,150,151].

## 6. Research Challenges and Future Directions

**Figure 10:**
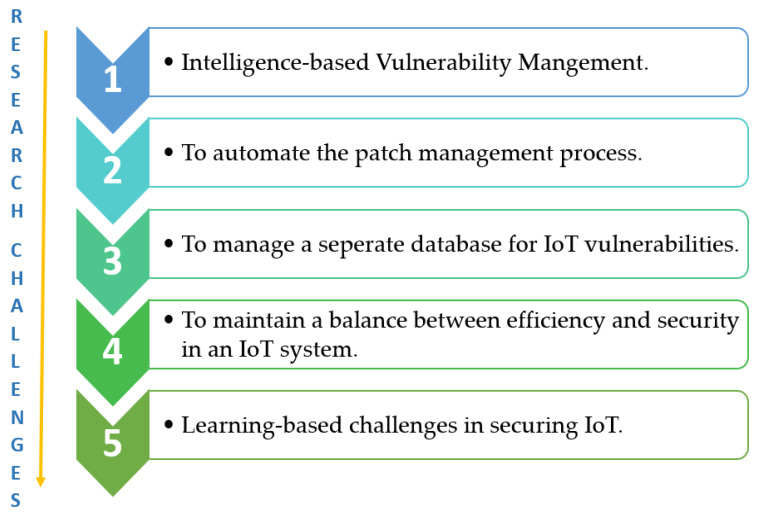
Emerging Challenges and Open Issues.

The expeditious advancement of IoT usage in multiple sectors brings security complications to the forefront. The tremendous volumes of research conducted in the past years still limit IoT to its nascent stage. The prime reason for the multiple challenges IoT is facing that limit its expansion is in the security zone. In this section, the emerging challenges which halt the IoT growth are discussed and pinpointed in Figure 10.

**i**  **Intelligence-based Vulnerability Management:** Firstly, the heterogeneity of the devices in the smart digitized world limits the automated detection and discovery of the vulnerabilities. Further, adding to this is the lightweight security requirement for their protection. These factors culminate the need to restructure the security analysis platform. The survey conducted in this paper also backs this restructuring by merging AI with IoT and presenting various solutions offered in this context. However, to further improvise the attack discovery, detection, and mitigation, some problems need to be confronted. These include a lack of real-time datasets. The datasets available for the research purpose do not reflect real-world attack scenarios and are often unbalanced. Further, the continuously changing functionalities of the networking environment require retraining of the system, thereby adding to the overhead.
**ii**  **To Automate the Patch Management Process:** The prime challenge to address the vulnerabilities in the smart devices is the lack of a single automated binary code patch generator that is functional across multiple platforms. The leading cause is the generation of devices by different manufacturers. Therefore, this prescribes their usability and prevents us from achieving an appropriate and feasible solution for the firmware patching. Further adding to this is the variable nature of the operating system and architectural patterns followed in the numerous devices. Thus, automatic patch generation requires a deep understanding of the entire mechanism, thereby making it a long-term security goal.
**iii**  **To manage a separate database for IoT vulnerabilities:** From the studied literature and growing attacks, it is seen that the general IoT devices with inherent known vulnerabilities are flooded to the market. These IoT nodes, in turn, act as a stepping stone for the adversaries to launch various attacks like Mirai, Hajime. Thus, to handle the insecure IoT devices, maintaining structured information about the exploits and known vulnerabilities in the smart environment would be of immense use. VARIoT is one such project working exclusively to develop a separate database for managing IoT vulnerabilities.

**i**  **To maintain a balance between Efficiency and Security in an IoT system:** In addition, a balance needs to be achieved between efficiency and data security. Due to the inverse nature, one often gets compromised. Therefore, incorporating ML and DL to the fog nodes must be explored in depth to the intelligence near the data sources to reduce the latency and the bandwidth. Though ML and DL can detect multiple attacks, still the challenge for mitigating all possible attacks persists. Therefore, supplementing the research further is required by exploring the incremental machine learning near the sources.
**ii**  **Learning-based challenges in securing IoT:** Machine Learning being known for extracting knowledge from the data were used for both malevolent and noble purposes. It is found that the potential adversaries make efficient use of these learning algorithms (machine learning and deep learning-based) to break the cryptographic secrets. For example, Recurrent Neural networks are being used by the authors for cryptanalysis. Furthermore, false data input feeds to the machine learning model result in improper functioning of the entire learning-based system. The problems of the oversampling, inadequate training dataset, and feature extraction are also a matter of concern in adding intelligence to smart environments.

## 7. Conclusions

The extensive study conducted in this research culminates in the various facets of IoT, beginning from the overview of the technology to the different architectural approaches. The outline is followed by an in-depth security analysis depicting a taxonomy of attacks, anomalies, and vulnerabilities. The technology has brought and will continue to bring numerous benefits to its pertinent implementation. However, the deep contemplation regarding the security aspects of it highlights the raising concerns in this sector. Thus, appropriate defense mechanisms like access control, IDS, and authentication are required to handle it. Due to the non-applicability of traditional security approaches (firewalls, antivirus) primarily because of low memory and computational constraints, other defense mechanisms like IDS have gained popularity. This paper highlights the numerous research efforts in the application of IDS based on the ML and DL algorithm as a security shield in this area. Additionally, the pros and cons of the various learning techniques are listed with their suitability towards different attacks conducted with critical analysis. Further, a case study highlighting the various facets of healthcare is also provided which further helps in understanding the practical implementation of IoT and learning-based security methods in real-world scenarios. The Smart Vehicular system and Smart Manufacturing systems are also explored in terms of their applications after being connected and the security challenges presented as a byproduct. Furthermore, after the extensive literature surveyed and presented, it is found that the critical issues namely automated patch management, intelligent vulnerability management system, and a separate depository for IoT vulnerabilities must be handled in hand for sustainable IoT. In the future, hybrid learning-based techniques will be explored to secure growing smart environments.

## Figures and Tables

**Table 2:** Prominent IoT architectures.

Author	Description
Bauer et al. [32]	IoT-A. An amalgamation of different IoT perspectives.
Atzori et al. [33]	The author has presented a SocialIoT-architecture based on the integration of IoT with the social networking concept.
Qin et al. [34]	The author presents SDN-based architecture for provisioning IoT with better quality-of-service, deployment, scalability, and context awareness.
Li et al. [35]	Mobility first (future internet architecture) mainly addresses the challenges concerning the usage of mobile phones as gateways and dealing with the security aspect of sensor data.
Singh et al. [36]	JDL (joint director of labs) based model for IoT architecture with the combination of semantic layer.
Cecchinel et al. [37]	Software architecture for collection of sensor-based data with cloud-based storage (sensor, sensor board, bridges, middleware)
Kraijak et al. [38]	5-layer architecture (perception, network, middleware, application, business)
Ray et al. [39]	It describes major IoT functional elements with multiple IoT architectures in different application areas.

**Table 3:** Attacks in IoT.

Nature of Attack	Description	Classification
Active attacks	These are performed mainly to carry out malicious acts against the system, thus affecting or disrupting the services for legitimate users. They hamper both the confidentiality and integrity of the system.	Dos (denial-of-service), DDOS (distributed denial of service), MITH (man-in-the-middle), Interruption, Alteration [48].
Passive attacks	These are performed mainly for gathering useful information without getting sensed, i.e., they do not disturb the communication.	Monitoring, Traffic Analysis, Eavesdropping, Node destruction/malfunction [49].
Physical layer attacks	These attacks try to tamper and exploit the devices making them the most vulnerable terminal of IoT.	Node tampering, Jamming, Replication [10].
Datalink layer attacks	These undertake the advantage of mac schemes to launch different attacks.	Collision, Dos, ARP spoofing, unfairness.
Network layer attacks	These attacks try to disrupt the communication between the source and the destination by playing with the packets.	Dos, Routing Attack, Sybil Attack, blackhole, spoofing, alteration.
Privacy threats	The capabilities of IoT allow it to launch acute attacks targetting the privacy of users.	Identification, profiling, tracking, linkage, inventory [50].
Software-based attacks	These attacks make use of third-party software to gain access to the system and cause destruction.	Virus, Trojan horse, Worms.
Side-channel attacks	These are hardware-based attack that uncovers the secret information like cryptographic keys to exploit the device.	Timing Analysis, Power Analysis.
Botnet attacks	These are a collection of infected devices (zombies) like printers, cameras, sensors, and similar smart devices, which launch large-scale DDOS attacks to compromise other intelligent devices. The principal components are command and control servers, along with the bots.	Mirai, Hydra, Bashlite, lua-bot, Aidra [51].
Protocol-based attacks	The attacks work against the connectivity protocols of IoT.	RFID-based (replay, tracking, killing tag) Bluetooth based (bluesnarfing, bluejacking, Dos), Zigbee Based (sniffing, replay, ZED sabotage attack) [52].

**Table 4:** Anomalies in IoT.

Type	Description
Point Anomaly	It is the most basic type of anomaly. One data point is abnormal in comparison to the rest of the data points.
Contextual Anomaly	It is a sophisticated type anomaly type where a data point is considered unusual in a specific context. For example, if any system accesses services at a particular time and if there is a sudden change in the background, i.e., time changes, it is considered abnormal.
Collective anomaly	Data points are anomalous w.r.t to the whole dataset or the entire services but not by themselves individually.

**Table 8:** Conclusions about learning approaches.

Ml And Dl Techniques	Advantages	Disadvantages	Suitability towards the Attacks
DT	Inherent feature selection, less preprocessing required, simple and easy to implement, can handle missing values, coupling with clustering decreases the processing time in misuse-based detection [29].	Large training time, large complexity, small alterations cause significant changes.	C4.0, C5.0 show very similar results to ANN in [110] with real IoT data. J48 shows a high affinity towards the DOS attack [111].
SVM	The Huge success rate in IDS, best for binary classification, requires small datasets for training, enhanced SVM shows better results in novel and real attacks.	Reveals its weakness in multiclass classification, massive consumption of memory, depends on the kernel function.	It is used in [9] for attack detection. Also useful in spoofing attacks, intrusions in access control [112], online outlier detection [113].
KNN	It has a Fast training phase and makes no assumptions about the data.	It requires abundant storage, expensive, depends on the value of K, and suffers from the dimensionality curse.	Mostly used in combination with other classifiers [48,107]. Useful for access control intrusion detection, malware.
RF	No feature selection, no overfitting problem, usually has the best accuracy.	Time-consuming because of the development of decision trees.	It has achieved 99% accuracy. for the DOS attack [106]. Useful for malware detection,link fault detection [83], access control.
NB	Robust towards the noise, simple and easy to implement	It cannot capture useful information because of the assumption of independence amongst the features.	Used in [49] for intrusion detection, access control.
ANN	Robust model and can handle non-linear data.	It suffers from overfitting, and the technique is time-consuming, selection of activation function is another overhead and estimating an appropriate number of units in each layer.	Very useful DOS attack detection [83,114].
RNN	Efficient modeling of time-series data	Difficulty in training, cannot remember very long sequences with Relu or tanh activation function [115].	Eavesdropping [107].
LSTM	Reduces a load of feature engineering, effective for unstructured datasets, can remember long sequences of attack patterns.	Difficult to train because of gigantic memory bandwidth requirements.	IoT malware [108], botnet activities, used in [116] for attack detection in fog networks.

## Data Availability

Data sharing not applicable. No new data were created or analyzed in this study. Data sharing is not applicable to this article.
